# Ecological Entomology: How Is Gibson’s Framework Useful?

**DOI:** 10.3390/insects12121075

**Published:** 2021-11-30

**Authors:** Aimie Berger Dauxère, Julien R. Serres, Gilles Montagne

**Affiliations:** The Institute of Movement Sciences, Aix Marseille University, CNRS, ISM, CEDEX 07, 13284 Marseille, France; julien.serres@univ-amu.fr (J.R.S.); gilles.montagne@univ-amu.fr (G.M.)

**Keywords:** optic flow, ecological approach, perception and action, low- and high order variables

## Abstract

**Simple Summary:**

Optic flow can be defined as a vector field of the apparent motion of objects, surfaces, and edges in a visual scene caused by the relative motion between an agent and the scene. In the last century Gibson developed the ecological approach to perception and action in which this optic flow provides an agent (i.e., human, insect, robot) all the information they need to navigate safely in cluttered environments. Gibson’s framework has already proved to be relevant in addressing issues related to the control of human displacement and by offering the robotics community a framework for carrying out new generations of studies. We would like to argue in this contribution that the ecological approach to perception and action, taken as a whole, provides powerful theoretical and methodological tools allowing the entomologist community to: (i) take a critical look at the research carried out to date, (ii) develop new experimental protocols, and (iii) raise new questions beyond the scope of current investigations. After a concise literature review about the perceptual control of displacement in insects, we will present the framework proposed by Gibson and suggest its added value for carrying out research in the field of entomology.

**Abstract:**

To date, numerous studies have demonstrated the fundamental role played by optic flow in the control of goal-directed displacement tasks in insects. Optic flow was first introduced by Gibson as part of their ecological approach to perception and action. While this theoretical approach (as a whole) has been demonstrated to be particularly suitable for the study of goal-directed displacements in humans, its usefulness in carrying out entomological field studies remains to be established. In this review we would like to demonstrate that the ecological approach to perception and action could be relevant for the entomologist community in their future investigations. This approach could provide a conceptual and methodological framework for the community in order to: (i) take a critical look at the research carried out to date, (ii) develop rigorous and innovative experimental protocols, and (iii) define scientific issues that push the boundaries of the current scientific field. After a concise literature review about the perceptual control of displacement in insects, we will present the framework proposed by Gibson and suggest its added value for carrying out research in the field of behavioral ecology in insects.

## 1. Perceptual Information Used in Flight Control in Insects: A Brief Review

**Figure 1:**
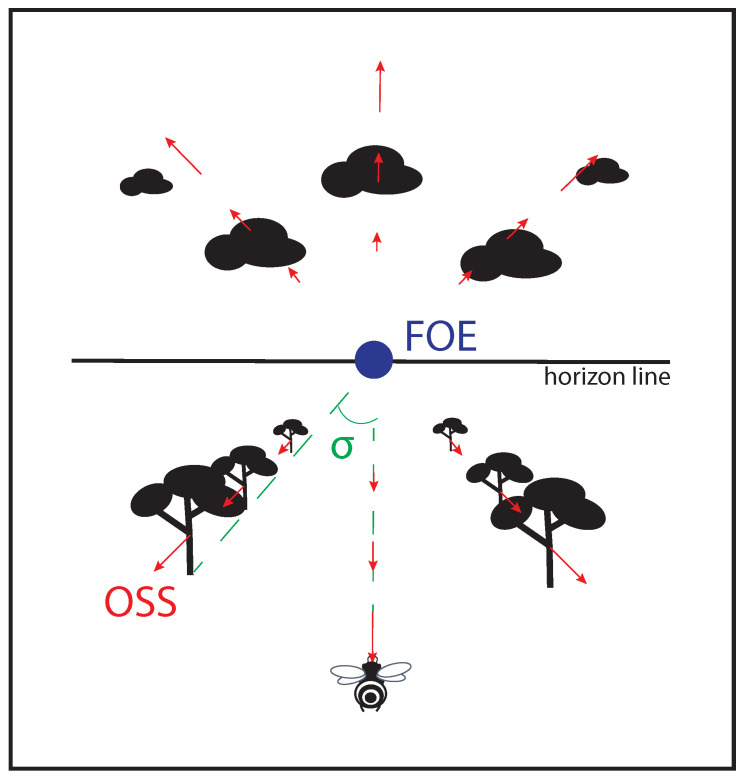
Non exhaustive optic flow variables perceived by a flying agent. Objects sliding on the optical sensor of an agent bear an optical velocity (OV) depending on the distance agent-object, and on the agent’s speed; focus of expansion (FOE) at the center of the visual field is the only point providing no OV; splay angle (σ) is the angle formed by the direction vector of the agent and one of the lines parallel to the movement joining the horizon.

Over the last eighty years, it has often been shown that flying insects rely on optic flow to perform various locomotion tasks [1,2,3,4,5]. Optic flow can be defined as a vector field of the apparent motion of objects, surfaces, and edges in a visual scene caused by the relative motion between an agent and the scene while being independent of the scene’s texture (see Figure 1 and [6,7,8]). Insects seem to be masters of spacial navigation due to their ability to extract perceptual information from optic flow in order to finely control their everyday locomotion tasks: terrain-following, centering, wall-following, speed adjustment, and landing (see review articles [3,4,5,9]).

- **Terrain-following task:** The ventral part of the optic flow is useful to follow the ground [3,10,11,12,13,14,15,16,17]. Srinivasan et al. [11] proposed “the image velocity of the ground is held approximately constant” to achieve a terrain-following behavior. Portelli et al. [13] proposed: “(1) honeybees reacted to a ventral optic flow perturbation by gradually restoring their ventral optic flow to the value they had previously perceived, (2) honeybees restored their ventral optic flow mainly by adjusting their flight height while keeping their airspeed relatively constant”. However, Straw et al. [18] “found that flies do not regulate altitude by maintaining a fixed value of optic flow beneath them”, but “*Drosophila* flies establish an altitude set point on the basis of nearby horizontal edges and tend to fly at the same height as such features”. A similar conclusion was drawn by David [10] about altitude regulation: “flies did not adjust the angular velocity of image movement that they held constant” for speed control purposes.
- **Centering and wall-following tasks:** The lateral/frontal parts of the optic flow are useful to center in a narrow corridor by balancing the lateral parts of the optic flow (in honeybees [3,19,20], in bumblebees [15,21], in flies [10,22], in hawkmoths [23]), or to follow a wall along a wide corridor by restoring the optic flow pattern from one side (in honeybees [19,20,24], in bumblebees [25]). Kirchner and Srinivasan [24] suggested “bees maintained equidistance by balancing the apparent angular velocities of the two walls, or, equivalently, the velocities of the retinal images in the two eyes”. Dyhr et al. [25] found a similar conclusion in bumblebees: “the centering response relies on a direct comparison of the optic flow from each eye providing a more accurate measure of the perceived differences”. Lecoeur et al. [15] found that “lateral position is controlled by balancing the maximum optic flow in the frontal visual field”. Stöckl et al. [23] found that “hawkmoths use a similar strategy for lateral position control to bees and flies in balancing the magnitude of translational optic flow perceived in both eyes”. Serres et al. [20] found that “bee follows the right or left wall by regulating whichever lateral optic flow (right or left) is greater”.
- **Speed adjustment task:** The bilateral (or bi-vertical) part of the optic flow is useful to adjust flight speed [3,21,26,27] (or [28]). Srinivasan et al. [26] showed that honeybees decrease their flight speed in a narrowing tunnel, and increase it as the tunnel widens. The authors of this study suggested that the visuomotor strategy consists of “holding constant the average image velocity as seen by the two eyes” without specifying any part of the visual field. A similar conclusion was drawn in flies [10,29]. Baird et al. [27] confirmed these results by manipulating the bilateral part of the optic flow and concluded that “honeybees regulate their flight speed by keeping the velocity of the image of the environment in their eye constant” by taking into account both the lateral and the ventral part of the optic flow. Portelli et al. [28] found in honeybees “that the ground speed decreased so as to maintain the larger of the two optic flow sums (”left plus right“ optic flows or ”ventral plus dorsal“ optic flows) constant according to whether the minimum cross-section was in the horizontal or vertical plane”.
- **Landing task:** The ventral part of the optic flow can also be regulated by honeybees to land [11,26]. Srinivasan et al. [11] concluded that honeybees “tend to hold the angular velocity of the image of the surface constant as they approach it” in order to adjust their height above a flat surface. Srinivasan et al. [11] also concluded that “the bee decelerates continuously and in such a way as to keep the projected time to touchdown constant as the surface is approached”. This “projected time” stringently means a time-to-contact (TTC). This procedure ensures the agent’s speed decreases proportionally with the distance to the ground, reaching a value near zero at touchdown [3,5,9].

As stated previously, most of the studies cited in this first section highlight the central role played by optic flow in the control of navigation tasks in insects. A close inspection of these studies demonstrates significant disparities between the terms used when referring to the same concepts or entities (see Table 1). It also reveals, in some cases, confusions between the perceptual support used by insects in the control process and the property of the environment to which it gives access. We would like to argue that these disparities and confusions reveal a need for some theoretical clarifications. To be fully useful by the entomologist community, the optic flow concept must be used in the same way as in its original theoretical framework, i.e., the ecological approach of perception action proposed by Gibson in the last century [6]. Gibson’s framework has already proved to be relevant in addressing issues related to the control of human displacement [30,31] and by offering the robotics community a framework for carrying out new generations of studies [32,33]. We would like to argue in this contribution that the ecological approach to perception and action, taken as a whole, provides powerful theoretical and methodological tools allowing the entomologist community to: (i) take a critical look at the research carried out to date, (ii) develop more rigorous and innovative experimental protocols, and (iii) define new scientific issues pushing the boundaries of the current research field.

## 2. Ecological Perception of the Visual World: Reminders of the Gibson’s Conceptual Framework

In the last century, through his ecological approach, Gibson provided a new conception of the links between perception and action. This new approach resolutely broke with the prevailing (cognitive) theories of perception, and has been made accessible through three major contributions (see [6,37,38,39]). Here, our ambition is to provide the reader with a synthesis of Gibson’s conceptual framework that could be of interest for conducting research in the domain of entomology.

### 2.1. A Conceptual Framework Anchored on a Double Postulate

As indicated above, Gibson took a controversial view of cognitive theories by building his new approach on a double postulate: the indivisible nature of the relationships (i) between an agent (either human, robot or insect) and the environment in which it operates, and (ii) between perception and action. According to the first postulate (i) the multiple interactions between a given agent and their environment shape (at both the evolutionary and individual scales) the perceptual-motor mechanisms used by the agent to move through their environment, so that the appropriate level of analysis to study these mechanisms is at the scale of the agent-environment system. According to the second postulate (ii) perception and action are part of the same cycle without any hierarchy. Contrary to the traditional view in which perception subserves action, Gibson described a circular relationship between perception and action in the sense that a movement gives rise to perceptual information which, in turn, allows movement to be adjusted and so on and so forth.

### 2.2. A Necessity: Redefining the Nature of (Perceptual) Stimulation

In accordance with ontological foundations (see Section 2.1) of the theoretical approach he wanted to promote, Gibson redefined the bases of perception [6]. Contrary to the shared belief of their time, he stated that sensory stimulation does not reside in frozen images, constituting approximate representations of the environment needing to be enriched through the implementation of high-level cognitive mechanisms. Instead, he considered that the information needed to control an action is directly available in the perceptual flows (e.g., optic, acoustic,…) without a need for enrichment. In summary, he did not agree with the proponents of cognitive approaches that supported a theory of poor sensorial input over a theory of rich perceptual input. The challenge for the agent would be to actively discover, in the perceptual flows the required perceptual information to fulfill the task at hand. Gibson spent a great deal of effort in describing and even formalizing the optic flow variables that could be used to control a given action (see [40,41] and Figure 1).

### 2.3. Where Is the Useful Information for Controlling the Action?

In the present review, we have chosen to focus on the *visual* control of displacement, based on optic flow. However, it is worth mentioning that the same reasoning holds for the other perceptual systems (see [42] for a relevant position paper on this topic). As stated previously, Gibson considered that the information required to carry out a given displacement is available in the perceptual flows, in our case the optic flow, which reach the agent. This optic flow corresponds to changes in the optic array following a displacement of the agent in the environment (also called global optic flow) or a displacement of objects within the environment relative to the agent (also called local optic flow). The optic flow generally combines local and global components without unduly complicating the issue of displacement control. The optic flow can be represented by a vector field characterizing (in amplitude and direction) the displacements of each point of the optic array following a displacement of the agent (Figure 1). An important point lies in the specificity of the flow in the sense that a given displacement gives rise to one, and only one optic flow. However, whatever the displacements produced, some styles of change in the optic flow are preserved (what Gibson called persistence over change). According to Gibson, the perceptual information useful for controlling the action lies precisely in these styles of change, called invariants [38], which precisely characterize the evolution of the agent-environment relation during the completion of a goal-directed displacement.

For example, when an agent moves forward, the optic flow expands radially from a stationary point, called the focus of expansion (FOE) (Figure 1), which coincides with the direction of travel. In the situation where an agent wants to control their direction of travel in a cluttered environment, control of the task consists of moving in such a way that the FOE coincides each time with the trajectory they want to follow [43,44] (provided they are able to detect the FOE). Let us now consider the case (called the *outfielder problem*) of an agent who wishes to intercept a moving target approaching along a parabolic flight. The only thing the agent has to do, assuming they are sensitive to the perceptual information, is to move appropriately so as to continuously cancel the optical vertical acceleration of the target [45,46]. Interestingly, this remarkable perceptual information reveals nothing about where and when the interception should occur, it just allows the agent to complete the task thanks to a strong coupling between perception and action.

### 2.4. The Need for Precise Terminology

Following the tenets of the ecological approach of perception and action briefly presented in the previous sections, perception is a matter of detecting, in the perceptual flow an invariant able to characterize the agent-environment system (AES). Given the fact that invariants are, by definition, unequivocally related to the state of the AES, detecting an invariant gives access to the state of the system; in other words, once *detected* the invariant *specifies* the state of the system (e.g., in Table 2). To come back to the previous examples, when the task requires directional control, detecting the location of the FOE tells the agent in which direction it is moving in the environment. When the agent’s task is to intercept a flying object, detecting the optical vertical acceleration of that object allows the agent to find out whether the current displacement speed is appropriate, i.e., will allow them to get to the right place at the right time. Following this line of thinking, the “perceptual problem” an agent must solve is to detect, in the perceptual flow, the appropriate invariant they need to control a given action. Although being beyond the scope of the current review Gibson also demonstrated through the *affordance hypothesis* [47] that moving across cluttered environments is a matter of perceiving the *passability* of gaps. More precisely, in the case of a high order variable being used to control self-displacement it must be scaled to either body or action capabilities. A given gap is said to be passable if it allows the body width to pass through it, or a moving prey is catchable provided acceleration capacities allow it. The readership interested in this area could refer to the convincing studies illustrating the validity of the affordance hypothesis in the domain of entomology [43,48,49,50].

**Figure 2:**
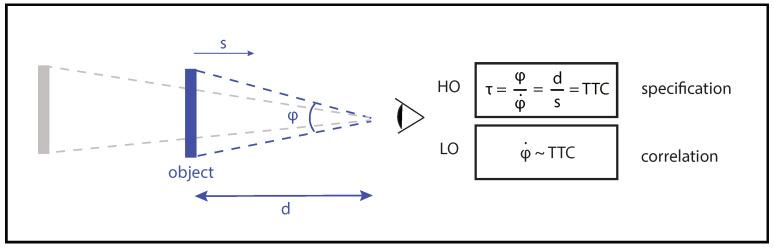
Difference between a high order (HO) variable and a low order (LO) variable in the example of object interception. A ball directly approaching the observation point is a paradigmatic example which can be used to illustrate the difference between low order and high order variables. τ, i.e., the relative rate of expansion of the ball, specifies the remaining time before collision (TTC) (provided the approach speed *s* is constant). The variable τ [58] is a high order variable in the sense that it gives a direct and accurate access to the remaining time before collision whatever the size, distance (*d*) and speed of the ball (*s*). The expansion rate of change ($\dot{\varphi }$) between two optical sizes can also be used to assess the time remaining before collision. Any object that approaches the observation point expands non-linearly with a sudden expansion increase just before collision (also called looming). However, the ball expansion pattern differs slightly depending on both ball size and ball speed so that the remaining time can only be approximated if this variable $\dot{\varphi }$ is used [55]. The expansion rate ($\dot{\varphi }$) is a low order variable in the sense that it allows the agent to approximate the time remaining before collision. Redrawn from Lee, 1976 [58].

The picture becomes a little more complex if we take into account the fact that perceptual flows also contain perceptual variables other than those called invariants. These variables, called heuristics, provide indications about the state of the AES without specifying it. A convenient terminology widely used in the literature to distinguish *heuristics* from *invariants* is to refer to them, respectively, as *low order* and *high order variables* (e.g., in [53]). To clarify, low order variables also called correlational variables, allow an approximation of the state of the system, while high order variables, also called specificational variables, allow a precise access to that state (Figure 2). Discussing the theoretical implications of the coexistence of these variables falls outside the scope of this article (see [54] for further information on this topic). As far as we are concerned, we consider that the coexistence of low and high order variables in the perceptual flows makes it possible to multiply the perceptual degrees of freedom available to the agent to carry out a task (e.g., in [55]). This multitude of perceptual degrees of freedom is particularly appealing if used in service of adaptive behavior. It potentially explains the extraordinary capacity of certain categories of agents to produce appropriate behavior in widely different environments, including impoverished ones [56]. It could also explain the changes of the perceptual variables used across training [57].

### 2.5. Identifying the Appropriate Experimental Agenda?

The experimental agenda that must be implemented when one wishes to take an interest in the issue of goal-directed displacement control within the framework of ecological psychology is as follows: analyzing task constraints, identifying the informational landscape, and specifically manipulating an optical variable.

*Analyzing task constraints.* Although it may initially seem trivial, the departure point of the research is defined by a close inspection of the constraints characterizing the task at the scale of the AES. Because optic flow contains potentially high order variables allowing access to the state of the AES, identifying the relevant property of the agent-environment system (PAES) is of paramount importance in a search of these high order variables. A couple of examples will clarify our point. In heading tasks, e.g., when making your way through a cluttered environment, the relevant PAES could reside in the direction of travel in relation to the surfaces of the environment. In interceptive tasks, i.e., when interacting with stationary or moving surfaces of the environment, the temporal proximity of the target could constitute the relevant PAES. In terrain following tasks, the PAES could reside in the flight height maintenance in reference to the ground.

*Identifying the informational landscape available.* Once the PAES has been identified, the information landscape allowing access to it can be described/formalized. Citing the three examples used in the previous section, the high order variables that could specify the state of the AES could be, respectively, the FOE (heading task), τ (interceptive task) or the rate of change of the optical velocity of the ground (terrain following task). As mentioned previously (see Section 2.3), the informational landscape available when performing a displacement can rarely be defined as a single high order variables and generally, for a given task, optic flow contains a great diversity of variables (both high order and low order) which can potentially be used (Figure 2). It is important to take these different variables into account in order to gain an integrated understanding of the underlying perceptual process.

**Figure 3:**
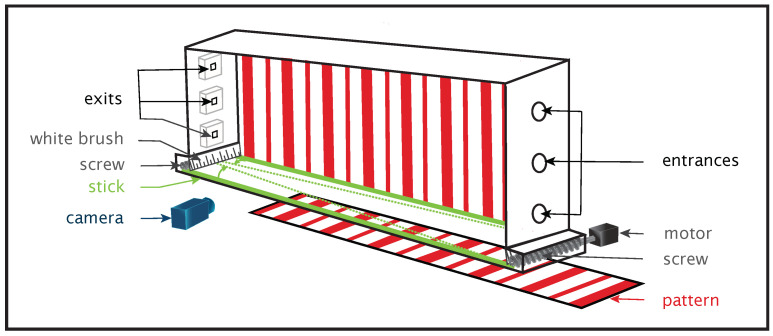
Experimental setup dedicated to high order variable manipulation. In an altitude-control task, two high order variables specify the AES state: optical velocity rate of change (OVRC) and splay angle rate of change (SARC). OV is the speed at which an object slides across the perceptual sensor of a moving agent, depending on agent’s speed and agent-object distance. Splay angle σ is the angle formed by agent’s direction vector and vanishing lines joining at the horizon (see Figure 1). When an agent loses altitude both OV and splay angle increase; the reverse is true in the case of an increase in altitude. This flight tunnel allows specific manipulations of these two variables; OVRC is easily accessible thanks to interchangeable textures: stripes or blank. Thus, OVRC can be *degraded* or *removed*. The SARC is provided by motorized sticks on the floor. The sticks can be made to converge giving rise to an (artificial) increase in the splay angle as generally observed when altitude is decreasing. The sticks can also diverge giving rise to an (artificial) decrease in the splay angle as generally observed when altitude is increasing. Thus, we can *falsify* the information provided by SARC about AES state. When OVRC is available but SARC is falsified, the two high order variables provide antagonist indications about AES state. Thus, the information provided by two high order variables can be *decorrelated*. The behavioral response of bees to artificial variations of SARC can then be quantified.

*Manipulating an optical variable.* Once the informational landscape available when performing a given task has been clearly identified, the ideal strategy is to implement an experimental stratagem allowing for a specific manipulation of an optical variable (Figure 3). This manipulation should, supposing the manipulated optical variable is used to control displacement, give rise to predictable behavioral changes. Some studies available in the literature provide elegant examples of experimental stratagems providing irrefutable proof of the use of a high order variable in the control of a goal-directed displacement task [51,59]. It is also true that many studies have described premature conclusions supporting the use of a specific optical variable while the results are at best compatible with the use of another one, but the causal function of the optical variable in the control process has not really been demonstrated.

## 3. How the Ecological Approach Allows a Better Understanding of the Processes Underlying Trajectory Control in Insects

### 3.1. The Ecological Framework as a Transversal Framework

One of the cornerstones of the ecological approach proposed by Gibson lies in the fact that it applies indifferently, whether we are interested in the behavior of humans, insects, or robots. It is this idea that appears in the background when Gibson refers to the concept of agents and considers that the perceptual information used to control a given displacement is available in the perceptual flows in which agents are immersed, not in the head of an agent after a cognitive post treatment. If we push this reasoning to the extreme, despite having different sensory modalities, humans or insects could detect the same high order variable(s) in the perceptual flows when they perform similar tasks.

### 3.2. New Reading of the Data from the Literature

While the literature contains numerous results which demonstrate the major role played by optic flow in the control of displacement in insects, the conceptual framework provided by Gibson (see Section 2) allows a more detailed analysis of the perceptual-motor processes implemented. As an example, when reviewing the different tasks mentioned in Section 1, several clarifications can be made.

- **Terrain-following tasks:** The studies mentioned [3,10,11,12,13,14,15,16,17] indicate that honeybees rely on the value of optic flow velocity (sometimes called global optic flow rate), a low order variable, to maintain their height. The key point in this particular task is that the high order variable is the OVRC, and not the value of the OV per se. Indeed, as OV is a ratio of speed over distance to the ground, further combinations of height and speed can provide the same OV value. In the case of forward displacement speed being constant, any variation in the OVRC “tells” the insect that the height is changing and requires a change in altitude. A close coupling between a high order variable and an action parameter allows the terrain-following task to be performed.
- **Centering task:** The centering behavior observed in many insect species [3,10,15,19,20,21,22,26] arises, in all probability, from the detection of a high order variable: motion parallax. Motion parallax corresponds to the OV gradient following a displacement of the agent in the environment [60]. This gradient makes it possible to locate the objects of the environment in relation to each other. When the two side walls have the same OV, they are equidistant from the observation point, i.e., the agent is moving along the center of the corridor. Equalizing the OV of the two walls guarantees the production of a centered displacement.
- **Speed adjustment task:** The studies reviewed in Section 1 seem to indicate that OVRC could be used as part of a safety principle. When a flight tunnel narrows or widens for a given displacement of the agent, the OV increases or decreases, respectively. Cancelling any change in OV despite changes in tunnel section gives rises to a safe behavior, i.e., a decrease in forward displacement speed when the tunnel narrows, and an increase in displacement speed when the tunnel widens.
- **Landing task:** Regardless of the fact that several high order variables can be used to control landing tasks, the study by Srinivasan [11] is interesting because it allows us to distinguish, as part of the control of a landing task, between a high order variable (tau: τ), the PAES it specifies (first order TTC) and how the high order variable can be used as part of a control strategy. Within the framework of the strategy described, maintaining the τ-value constant is a sufficient condition for zeroing velocity displacement as the surface is approached.

### 3.3. The Challenge of Determining Which Variable Is Used and How It Is Used

As Gibson’s framework is not species-specific, it is easily adaptable to carry out research in insect behavior. It enables us to think in terms of the available informational landscape to better design experimental protocols and setups. It also allows us to focus on the variable(s) leading to the most parsimonious strategy when performing a goal-directed task. Keeping these ideas in mind, a new reading of research focusing on the role of optic flow in insects becomes possible.

When examining studies focusing on the identification of perceptual variables involved in flight control, it is essential to clearly differentiate low order and high order variables. Perceptual strategies relying on high order variables could be prioritized by insects given that they are based on a precise access to the state of the AES and so provide robust strategies leading to safe behaviors. However, and counterintuitively, in some cases low order variables are preferred to high order variables [18,35]. Straw et al. studied visual control of altitude in flying by *Drosophila melanogaster* though tunnels which allowed an easy manipulation of lines on the side walls, whose height could be changed during and between trials. Despite the fact that the ground was textured so as to supply easy access to optic flow velocity (and its rate of change), flying *Drosophila* preferred to follow these prominent lines. At first glance this result may seem surprising, as flying *Drosophila* seem to prefer using a low order variable (line tracking) instead of a high order variable (the rate of change in optic flow velocity). When performing this type of task, the line provides a low order perceptual variable as line following does not in any way guarantee that altitude will be maintained. This perceptual strategy can be extremely dangerous in an ecological context but allows the flying *Drosophila* to perform the task easily in the highly constrained environment used in the experiment. While demonstrating the extraordinary flexibility of insects, this result should urge caution; the results obtained in a very constrained environmental setup are not necessarily generalizable as such to other ecological contexts. That is the reason why 3D environments should be preferred over 2D environments when trying to identify the perceptual variables underlying the control of insects flight. Insects’ perceptual systems have evolved and adapted through interactions with their 3D environments so that the 3D properties of the environment should be preserved as much as possible [15,48,49,61,62].

The issues related to the identification of the perceptual variables involved in the control of landing tasks by insects have also deserved a lot of interest in recent years [36,63,64,65,66,67,68]. All these studies highlighted the fact that the expansion pattern of the surface on which the insect wishes to land is particularly relevant. Now, once again, a precise formalization of the perceptual variable in question and of the way in which it could be used in the control process is required. These clarifications are all the more necessary because several distinct perceptual variables rely on the expansion optical pattern of the landing surface, each serving potentially different purposes. As an example, a given action (e.g., leg extension) can be initiated when either the expansion rate ($\dot{\varphi }$) or the relative rate of expansion (φ/$\dot{\varphi }$) of the landing surface reaches a critical value (Figure 2). These perceptual strategies rely on the use of either a low order variable ($\dot{\varphi }$) or a high order variable (φ/$\dot{\varphi }$) and allow specific experimental predictions to be made depending on both the approach speed of the insect [36] and the size of the landing surface. It is worth noting that the function of the expansion pattern of the landing surface is not limited to the control of the initiation of a discrete event (i.e., leg extension in the previous example). It can also be used as part of the continuous control of deceleration when approaching the landing surface. Several studies have indicated that the rate of change of τ over time ($\dot{\tau }$) is a particularly relevant high order perceptual variable [11]. The only thing the insect has to do to land safely on a surface is to decelerate so as to keep the $\dot{\tau }$ around a value of −0.5. This strategy ensures that the insect stops at the moment of contact with the surface. Here too, the use of this high order variable allows precise predictions to be made concerning the kinematics of the landing phase.

### 3.4. The Challenge of Understanding the Whole Informational Landscape

One of the lessons that can be learned from Gibson’s contributions, is that all the studies focusing on the perceptual control of goal-directed behavior have everything to gain by taking into account the whole informational landscape available to the agent when performing the task. The study by Baird et al. [34] is prototypical of the added value associated with this approach. The study was designed to identify the optic variables underlying the control of centering behavior in *Megalopta genalis*. The insects were trained to move along a hallway whose walls could provide either a strong optical velocity (thanks to a checkerboard pattern) or weak optical velocity (thanks to horizontal stripes). Contrary to the authors’ expectations, *Megalopta genalis* was shown to exhibit more variance in their centering response in the presence of checkerboard patterns in comparison with the other condition. Although this result could initially appear counterintuitive, a deeper analysis of the informational landscape provided to the insects in the two conditions sheds a different light on this issue. While the checkerboard pattern allows a centering behavior based on the use of motion parallax (see Section 3.2), horizontal stripes facilitate access to the splay angle (see Figure 1 and Figure 3). Not only has a strategy based on the equalization of the splay angles subtended by the two walls been showed to elicit centering behavior in humans [51], but this strategy has also been demonstrated to be more robust than the one based on the use of motion parallax. The study by Baird et al. [34] could benefit from the framework proposed by Gibson.

To conclude this section, we would like to clarify that our objective is certainly not to denigrate the work carried out to date, but to try to show how it could be enhanced through a consideration of the framework provided by Gibson. We believe that for each experiment carried out, taking into consideration the whole informational landscape and questioning the status of the variables identified in close connection with the ecological framework, would allow the researchers to be ideally placed to identify the underlying perceptual processes. High order variables can form the basis of robust strategies adaptable to various environments, while strategies based on low order variables may appear to be more fragile. Unsurprisingly, insects having evolved in a wide variety of environments, use this kind of robust strategy (see Section 4.2). Programming new experiments designed to examine to what extent these strategies could be shared by different species would be a very exciting challenge.

## 4. Opening Up New Avenues for the Community of Entomologists

### 4.1. How This Informational Abundance Can Be Used

As already mentioned in this review, one of the most striking things is the multitude of perceptual degrees of freedom available for a given task (see Section 2.4 for further information). The question that naturally arises is to discover how an agent manages this informational abundance. For a given task both low order and high order variables coexist, but also several high order variables are generally available allowing each variable a precise access to the AES. This observation leads us to question the nature of the perceptual processes implemented. More precisely, does the agent select the most appropriate optical variable to complete the task or do they use several optical variables jointly as part of a single process?

This issue has been addressed by Duchon and Warren [51] in an experiment carried out in humans. The authors investigated the perceptual support used by the participants to produce a centered behavior when moving through a virtual corridor. They first identified three high order variables which could each allow a centered behavior. Very clever manipulations allowed the authors to analyze the behavior of humans when these high order variables were either removed or biased. The main results indicated that three high order variables are used jointly in the control process; this led the authors to formalize a law of control: (i) linking the three high order variables to a movement parameter, and (ii) establishing a weight regime between the three variables. When several perceptual variables are simultaneously used, some combinations of processes come into play which allow priorities to be established among them [69].

We may wonder if there is any reason to believe that evolution has caused insects to develop perceptual processes distinct from those of humans. In other words, while many experiments provide some fuel for the idea that humans use several perceptual variables, when available, in combination [30,70,71], do insects proceed differently? Interestingly Straw et al. [18] provided results in agreement with the idea that flying insects (*Drosophila*) would also use a combination of perceptual variables in the control of altitude. Although the terminology used by the authors is based on a theoretical framework other than Gibson’s (the authors evoke “a combination of three sensory-motor reflexes”), the results of their study support the thesis of the joint use of different perceptual variables by the insect.

The idea of a combination of different perceptual variables is very attractive because it gives a certain robustness to the perceptual process. The agent can theoretically adapt its flight to a degraded environment and/or to a momentary absence of certain variables if other variables are available. This new conception of the perceptual process paves the way for a new generation of studies focusing on the underlying combination process. In the case of two high order variables being available, is there necessarily a priority between them and, if so, on what basis? Does the nature of the environment in which the agent is operating affect the prioritization of the perceptual variables used? In any case, it will be necessary in the future to focus on the combination processes in order to identify the prevailing principles and ideally to formalize them.

### 4.2. Adaptation and Learning: Two Processes at Serving Behavioral Flexibility

The abundance of perceptual variables mentioned in the previous section provides agents with perceptual degrees of freedom potentially allowing them to rapidly adapt to unexpected changes in their surroundings, and also to optimize their behavior across repetitions in the longer term. The two processes (adaptation and learning) could be interesting in the domain of entomology, considering the extraordinary ability of insects to move safely in cluttered environments. Here again, to our knowledge, these processes have received little attention in the domain of visual ecology in insects, in comparison with the colossal number of studies dedicated to these processes in humans.

**Figure 4:**
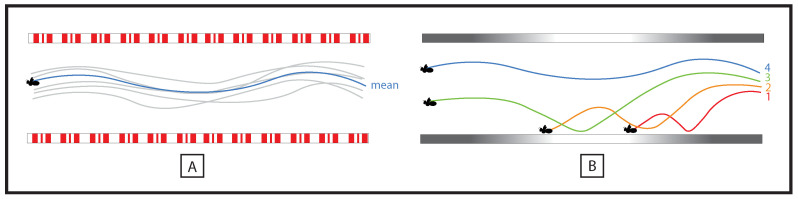
How to study the adaptive process in insects. Insects have been shown to be able to go through tunnels provided the tunnel surfaces (floor and ceiling in particular) are textured and optic flow is available (**A**). A recent study has shown that when a set of mirrors replaces the floor and the ceiling bees crash irremediably [16,17]. This raises the question of the extent to which bees are able to adapt to this strong perturbation through iterations. Not only would the kinetics of this adaptation be worth considering (**B**), but also the conditions necessary for the appearance of this adaptation could be identified. An experiment could consist of analyzing bees’ trajectories in the control condition (**A**) and then to examine how bees adapt to the strong perturbation provided by the two mirrors and to what extent flight success could be restored through repetitions (**B**).

The studies designed to examine the adaptation process in humans generally used a dedicated three-step methodology designed to: (i) examine the behavior in a control condition, (ii) examine the kinetics of the adaptation process following a perturbation, (iii) return to the control condition so as to assess the presence of a post-effect. Examining the kinetics of the adaptation process in insects following, as an example, a severe degradation of the perceptual support available could be worth considering (Figure 4). This kind of study would make it possible to determine the minimal informational context from which a navigational task could be performed or restored.

**Figure 5:**
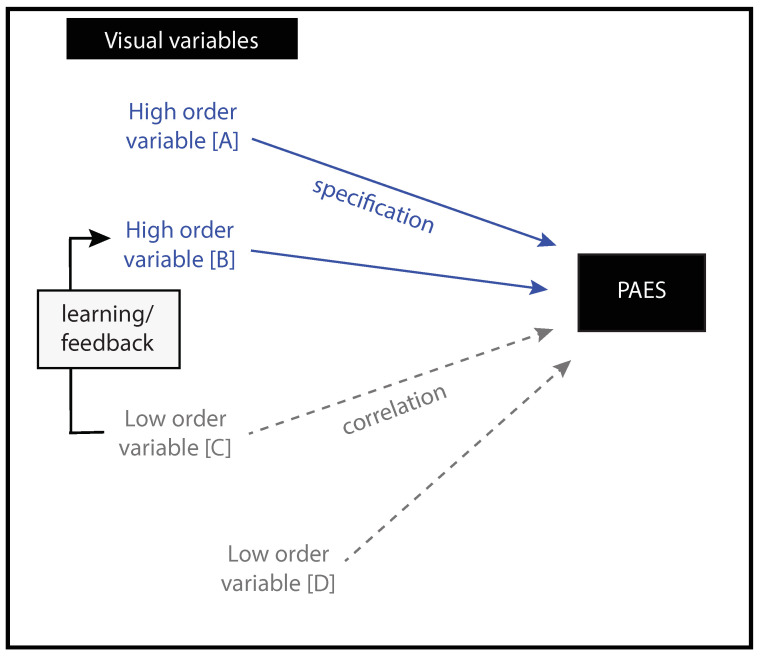
Representation of the informational landscape potentially accessible to agents. For a given task, several variables (either high order [A,B] or low order [C,D] variables) provide a more or less precise access to the relevant Property of the Agent-Environment System (PAES). As illustrated in the figure above, in humans perceptual learning has been described as the education of attention towards relevant perceptual variables, provided that feedback is available. Generally during learning, low order variables are abandoned (e.g., the variable [C]) in favor of high order variables (e.g., the variable [B]) as the use of a precise access to the PAES in a given task maximizes the chances of success.

However, it seems to us that particular attention should be paid to the study of the processes underlying the (perceptual) learning of navigational tasks in insects. According to the tenets of ecological psychology, perceptual learning is a matter of educating the attention of the agent, through training, towards the relevant perceptual variable. A number of experiments in humans have demonstrated that learning is accompanied by a change in the perceptual variable(s) used preferentially, with a shift from lower order variable(s) to higher order ones [57,72] (Figure 5). The central point here lies in the fact that learning is thought to involve implicit processes, in the sense that the reliability of a given perceptual variable is assessed by the agent on the basis of successive trials, provided that feedback relative to the result is available. For a given agent, learning is about discovering, in optic flow, the most reliable perceptual variable(s) to accomplish a given task. In this context, the question is to know whether similar perceptual learning principles can be described in insects or if distinct pressures in terms of species evolution (humans vs. insects) have given rise to different perceptual organizations with respect to learning.

## 5. Conclusions and Perspectives

The stated ambition of this review is to promote the conceptual framework provided by Gibson [38] to address issues related to the perceptual control of goal-directed displacements in insects. We have shown how the use of this framework could allow the entomologist community to have a more precise idea of the different perceptual degrees of freedom available, potentially useful for insects, in perceptual visual flows. The difference in the status of these perceptual degrees of freedom (low order vs. high order variables) would be worth considering in future studies. The ecological approach of perception and action also provides a very convenient methodological framework with a clear experimental agenda which would help to guide the researcher’s approach. Last but not the least, this approach would allow a renewal of questions and guide future research towards new scientific challenges. We hope our contribution could, to some extent, pave the way for a future generation of ‘ecologically inspired’ studies that would participate in the debate of ideas among researchers in the entomologist community.

## Figures and Tables

**Table 1:** Variability of denominations used in the entomologist community to describe perceptual information.

Task	Denomination of the Perceptual Information in Entomology	Species
Centering and wall following	(lateral) “image angular velocity” in [34] “horizontal optic flow cues” in [34] “speed of retinal image motion” in [26] “apparent angular speed” in [26] “lateral optic flow” in [20] “the magnitude of translational optic flow perceived in both eyes” in [23] “translational optic flow cues” in [35]	*Apis mellifera*, *Bombus terrestris*, *Megalopta genalis*, *Macroglossum stellatarum*
Speed adjustment	(lateral) “image angular velocity” in [11,27] “optic flow cues in the lateral visual field” in [27] “velocity of the perceived image motion” in [27] “rate of optic flow” in [21,27] “image motion signal” in [27] “optic flow cues” in [27] “apparent velocity of the surrounding environment” in [26] “apparent movement of the surrounding patterns relative to themselves” in [10] “retinal slip speed” in [29]	*Apis mellifera*, *Bombus terrestris*, *Drosophila hydei*, *Drosophila melanogaster*
Terrain following	“ventral optic flow” in [13] “apparent (ventral) speed of image” in [26] “image angular velocity” in [27] “optic flow cues in the ventral region of the visual speed” in [27] “rate of optic flow” in [27] “perceived image velocity of motion of the image” in [27]	*Apis mellifera*
Landing on vertical surface	“tau: apparent rate of expansion of the image” in [11] “magnitude of optic flow” in [36] “speed of image motion on the retina” in [36]	*Apis mellifera*
Landing on horizontal surface	“angular velocity of the image” in [11,26]	*Apis mellifera*
Heading	“apparent movement” in [1] “retinal image displacement” in [1]	*Aëdes aegipty*

**Table 2:** Task relevant high order optical variables and corresponding system agent-environment properties. For a given task, a high order visual variable provides direct access to a property of the agent-environment system (PAES).

Task	High Order Variable	Relevant PAES
Centering in a narrow corridor	motion parallax [51]	distance to center of corridor
Maintaining speed	OVRC [27,28]	speed maintenance
Maintaining altitude	splay angle rate of change (SARC) [52], OVRC [13,27]	altitude maintenance
Landing on a vertical surface	τ $\dot{\tau }$ [36]	Time-to-Contact (TTC), relevance of current deceleration
Landing on a horizontal surface	OVRC [11]	altitude change
Heading	Focus Of Expansion (FOE) [30]	direction of agent’s displacement in relation to environment
Object interception	bearing angle rate of change [31], optical velocity rate of change [46]	adequacy of the current velocity in relation to the object’s trajectory

## Data Availability

Not applicable.

## Abbreviations

AES	Agent-Environment System
FOE	Focus of Optical Expansion
OV	Optical Velocity
OVRC	Optical Velocity Rate of Change
PAES	Property of the Agent-Environment System
SARC	Splay Angle Rate of Change
TTC	Time-To-Contact
